# Predicting Long-Term Care Demands and Optimal Strategies for Older Persons with Dementia in China to 2040

**DOI:** 10.1093/geroni/igaf122.2228

**Published:** 2025-12-31

**Authors:** Peijia Zhu, Qifan Yang, Jie Chen, Liangwen Zhang, Ya Fang

**Affiliations:** Xiamen University, Xiamen, Fujian, China (People’s Republic); Xiamen University, Xiamen, Fujian, China (People’s Republic); Xiamen University, Xiamen, Fujian, China (People’s Republic); Xiamen University, Xiamen, Fujian, China (People’s Republic); Xiamen University, Xiamen, Fujian, China (People’s Republic)

## Abstract

**Objective:**

Limited evidence exists on the future trajectory of long-term care (LTC) demands for older persons with dementia in China. This study aims to quantify projected changes in the dementia population from 2020 to 2040 and provide evidence for national dementia LTC planning and informal care support.

**Methods:**

Using multi-source data (CLHLS 2011/2014/2018, UN projections, official statistics, surveys, interviews), we developed and validated a Markov model with age, gender, and urban-rural differences, with a 1-year cycle. Using dementia transition probabilities, the model projected trends in the dementia population from 2020 to 2040. Scenario analyses (low and high standards) were conducted to explore demand characteristics in care workforce and costs from both formal and informal care perspectives.

**Results:**

The dementia population is projected to rise by 51.2%, from 19.73 million in 2020 to 29.83 million in 2040, with mild dementia at 60.16%. Informal care workforce remains dominant, gaps range from 23.34 million (low standard) to 38.33 million (high standard) by 2040. Dementia LTC costs as a share of GDP are notable (0.26%-0.64%), with formal care costs exceeding informal care, the main driver is increasing new dementia cases due to population aging.

**Conclusion:**

Informal care plays a critical role in dementia LTC and needs to be supplemented with diverse formal care services. Additionally, expanding community-based screening and intervention for high-risk populations with mild dementia, along with providing tiered support and corresponding subsidies, can ensure that resources are precisely allocated to meet the care needs across different stages of dementia.

